# Multifinality in pathways from early ecological adversity to children’s future self-regulation: Elucidating mechanisms, moderators, and their developmental timing

**DOI:** 10.1017/S0954579425000148

**Published:** 2025-03-21

**Authors:** Juyoung Kim, Grazyna Kochanska

**Affiliations:** Department of Psychological and Brain Sciences, The University of Iowa, USA

**Keywords:** ecological adversity, internal working model, power assertion, self-regulation

## Abstract

Detrimental impacts of early ecological adversity on children’s development are known, but our understanding of their mechanisms and factors contributing to multifinality of developmental trajectories triggered by adversity is incomplete. We examined longitudinal pathways from ecological adversity parents experienced when children were infants, measured as a cumulative index of fine-grained scores on several ecological risks, to children’s future self-regulation (SR) in 200 U.S. Midwestern community families (96 girls). Parents’ observed power-assertive styles were modeled as mediators, and their negative internal working models (IWMs) of the child, coded from interviews – as moderators. Both were assessed twice, at 16 months and at 3 years, to inform our understanding of their developmental timing. Children’s SR was reported by parents and observed at 4.5 years. Path analyses revealed moderated mediation in mother-child relationships: A path from higher early ecological adversity to elevated power assertion to children’s poorer SR was significant only for mothers with highly negative IWMs of the child. Maternal negative IWMs assessed early, at 16 months, moderated the link between ecological adversity and power assertion. Once elevated, maternal power assertion was stable through age 3 and not moderated by IWM at age 3. There were no significant effects in father-child relationships.

## Introduction

Learning to internally regulate behavior is a key developmental task in early childhood. Voluminous research has examined top-down self-regulation (SR), sometimes referred to as effortful control, self-control, inhibitory control, or executive function, encompassing processes of modulating thoughts, affects, behaviors, or attention through the deliberate use of specific skills to guide goal-directed activities over time and across various contexts (Karoly, 1993; Kopp, 1982; Nigg, 2017; Rothbart & Bates, 2006). That research has robustly demonstrated that SR is concurrently and longitudinally associated with a broad range of adjustment measures: interpersonal relationships, school readiness, academic achievement, behavioral adjustment, and physical and mental health (e.g., Eisenberg et al., 2024; Kochanska et al., 2000, 2001; Kostyrka-Allchorne et al., 2020; McClelland et al., 2007, 2018; Moffitt et al., 2011, 2013; Rademacher & Koglin, 2019; Robson et al., 2020; Spiegel et al., 2021; Yang et al., 2022). Thus, identifying antecedents and factors that account for individual differences in children’s SR is a critical goal, essential for both basic research and translational efforts to foster adaptive and healthy development and prevent negative outcomes.

Broadly accepted ecological perspectives have long portrayed children’s development as best understood when considered in a rich, multilayered context (Belsky, 1984; Bronfenbrenner & Morris, 2007; Cicchetti & Rogosch, 2002; Taraban & Shaw, 2018). Challenges in families’ environments, especially ecological adversity impinging on parents at the time when they assume the caregiving role, can substantially undermine children’s future adaptation trajectories. Ecological adversity can refer to a single dimension of the early environment (e.g., poverty, parental young age, low education, or stressful events) or it may represent a combination of multiple dimensions. Many researchers have advocated for the latter approach to achieve a more comprehensive understanding of contextual effects on child development (Appleyard et al., 2005; Deater-Deckard et al., 1998; Evans et al., 2013; Kraemer et al., 2005; Sameroff, 1998). Empirical evidence has shown that a confluence of multiple risk factors is especially detrimental (Evans, 2003; Roy & Raver, 2014; Sameroff et al., 1987). Consequently, we defined and measured ecological adversity as a construct that integrates cumulative risks associated with various demographic and environmental sources; we then investigated its developmental implications for children’s future SR.

According to the bioecological model (Bronfenbrenner & Morris, 2007), child development occurs in intricate interactions between individuals and environments through proximal processes that vary as a function of a person, immediate and distal environmental contexts, and time. In this perspective, the pathway from early ecological adversity to children’s SR can be understood as multi-faceted, multi-layered processes influenced by distal and proximal environmental factors both directly and indirectly.

Children’s future SR can be directly influenced by ecological adversity early in their lives. A large body of research has highlighted negative implications of early ecological adversity on children’s developing SR or related outcomes, such as behavioral problems (e.g., Gach et al., 2018; Lengua et al., 2014; Wade et al., 2022; Wallander et al., 2019; Wanless et al., 2011), and linked exposure to environmental risks to the impact on brain networks and functions related to SR (Blair, 2010; Cornwell et al., 2023; Gee & Cohodes, 2023; Pechtel & Pizzagalli, 2011).

SR can also be indirectly affected within person-context interactions (Liew et al., 2023). A large body of evidence has documented that negative impacts of early ecological risks on later developmental outcomes, including SR, were largely accounted for by the effects of those risks on parenting, which is the most prominent proximal factor early in life (Abidin, 1992; Belsky et al., 2007; Blair & Raver, 2012; Hackman et al., 2015; Lengua et al., 2014; Mistry et al., 2010; Scannell, 2020; Trentacosta et al., 2008). Ecological adversity can overwhelm parents and disrupt the quality of caregiving, ultimately exacerbating children’s difficulties in regulating their emotions and behaviors. However, most studies have primarily focused on adversity’s detrimental impacts on positive parenting, including warmth, responsiveness, availability, autonomy support, and appropriate scaffolding and structuring. While informative, this approach is incomplete, as it underestimates adversity’s exacerbating impact on negative aspects of parenting, which have also been implicated as factors that undermine children’s SR, for example, intrusiveness (Geeraerts et al., 2021), or harsh, coercive, and power-assertive control (e.g., Kochanska & Knaack, 2003).

Despite negative associations among ecological adversity, parenting, and developmental outcomes, only a handful of studies have examined the entire path from adversity to negative parenting to SR. For instance, Rhoades et al. (2011) found significant indirect relations between environmental risk in infancy and executive function at age 3 years through both positive and negative mother-child interaction, but this study examined separate paths from each risk indicator. Another study with cumulative risk as a predictor found significant mediation from cumulative risk in infancy to behavior outcomes and executive function at age 3 years through both negative (harsh and controlling) and positive (sensitive and responsive) parenting (FLP Key Investigators, 2013).

Importantly, although the effects of ecological adversity on dysfunctional parenting have been well documented, not all parents are uniformly affected. As with all types of adversity and stress, there is great heterogeneity in human response (Rutter, 2013); many parents show resilience and ability to function adaptively in the parenting role despite challenging circumstances. Multiple factors have been implicated as determinants of parents’ varying ability to function effectively despite adversity (e.g., Löchner et al., 2024). Understanding the diversity in cascading developmental processes (i.e., multifinality) – or moderators of the paths triggered by early ecological adversity – is an important research goal that remains at the center of developmental psychopathology (Cicchetti & Rogosch, 2002; see also the recent Special Issue of *Development and Psychopathology*, Masten et al., 2023).

Recently, researchers have increasingly focused on parental mentalization processes as a set of critically important inner resources that may affect trajectories of parenting, parent-child relationships, and child developmental outcomes, including SR. Parents’ mentalization, often rooted in their own relationship histories, encompasses both implicit and explicit components (relational schemas, reflective functioning, mind-mindedness, perceptions, expectations, and attributions regarding their child). That exponentially growing research, often informed by the attachment perspective and its constructs of parents’ internal working models (IWMs) of the child, has demonstrated a key role such mental representations play in parents’ relationships with their children (e.g., An et al., 2022; Camoirano, 2017; Dykas & Cassidy, 2011; Garon-Bissonnette et al., 2024; Humphreys et al., 2024; Katznelson, 2013; Kochanska et al., 2019; Luyten et al., 2020; McMahon & Bernier, 2017; Sharp & Fonagy, 2008; Sher-Censor, 2015; Weston et al., 2017).

Mentalization processes can also influence how parents cope with stress (Buttitta et al., 2019; Camoirano, 2017; Park et al., 2018; Snyder et al., 2005). Research has supported a model that focused on parental IWMs as moderators of the association between child difficulty – a common source of parenting stress – and the parent’s harsh, power-assertive discipline (An & Kochanska, 2022b, Kochanska & An, 2024a, 2024b; Kochanska et al., 2019). Parents with negative IWMs perceive their child’s difficult behaviors as intentional and deliberate, increasing the risks of their resorting to power-assertive or harsh parenting.

In the current work, we expanded that model to examine whether parents’ negative IWMs of their children can moderate the effects of early ecological adversity on their parenting and future developmental trajectories. As mentioned earlier, developmental sequelae of ecological adversity can be highly divergent, leading to diverse outcomes depending on moderating factors, as highlighted in the concept of multifinality (Cicchetti & Rogosch, 2002). Parental mentalization or IWMs of their children can shape how parents interpret and respond to environmental stressors, potentially exacerbating or mitigating the adverse effects of ecological risks (Narayan et al., 2015). Of note, for parents coping with high adversity, the effects of mentalizing have been especially pronounced (Engbretson et al., 2023; Meins et al., 2013, 2019; Suchman et al., 2017; Wendelboe et al., 2024). Humphreys and colleagues (2024) characterized parental representations of the child as the key factors linking the caregiving context and parenting, with cascading effects on child outcomes, and crucial for understanding resilience. Based on the earlier research, we expected the paths from early ecological adversity to dysfunctional parenting to be stronger for parents who had more negative IWMs of their children.

Bioecological and developmental psychopathology models presented above align also with adult research on transdiagnostic models of the origins of psychopathology, with ecological adversity, or environmental context, conceived as a distal risk factor. Nolen-Hoeksema and Watkins (2011) proposed that distal risk factors, such as environmental context, predict proximal risk factors, which in turn lead to future disorders, or psychopathology outcomes. The associations between proximal risk factors and disorders can be moderated by a number of variables, accounting for divergent developmental trajectories. Although Nolen-Hoeksema and Watkins (2011) conceptualized proximal risk factors mediating between distal risk factors and psychopathology as within-person characteristics, we believe that their model can be adapted to research like ours, which aims to connect features of the distal environment, or adverse ecology, the parent-child relationship, and child outcomes (poor SR). We see dysfunctional parenting as a proximal factor that mediates the path from the distal factor (ecological adversity) to child poor SR.

In addition, we examined if parental negative IWMs of the child can also serve as a moderator of the stability of parental power assertion over time. To our knowledge, few, if any, studies have yet addressed this question. Parenting styles are often assumed – and found – to be modestly to moderately stable over time and typically modeled as autoregressive paths in studies where their measures have been obtained repeatedly (e.g., Dahl & Chan, 2017; Dallaire & Weinraub, 2005; Hoeve et al., 2008; Holden & Miller, 1999; Kochanska, 1990; Kochanska et al., 2003; Loeber et al., 2000; Teuber et al., 2022; Verhoeven et al., 2007; Zhang et al., 2017), across multiple cultures (e.g., Putnick et al., 2018). Of note, in a study with high-risk families, negative parenting profiles, such as harsh-intrusive and detached parenting, were more consistent over one year than positive parenting styles (Owen et al., 2023). However, many of those studies were limited due to their reliance on parental or child self-report. This could make it hard to distinguish the stability of actual parenting from that of measurements, often reported by the same informant, and likely stable. Thus, assessing parenting behaviors in naturalistic settings may provide more accurate information on the stability of parenting. Studies that have relied on behavioral measures of parenting have often demonstrated only moderate rank-order stability over time, suggesting that changes in parenting over time, including power-assertive control, are also quite likely (Dahl & Chan, 2017; Kochanska et al., 2003).

We explored whether the parent’s negative IWM of the child can account for both elevating and maintaining dysfunctional power-assertive parenting over time. Parents with negative schemas regarding their children may be more inclined to see power assertion as effective in controlling children’s behaviors and to continue to rely on it consistently. Research on abusive mothers, who often perceive their children in a negative light, has shown that, in comparison to non-abusive mothers, they resorted to power assertion much more often, regardless of the discipline situation (Trickett & Kuczynski, 1986). In contrast, parents with less negative schemas may use diverse strategies and be more flexible and open to adapting their parenting to changing circumstances, such as the child’s developmental level, the type of misbehavior being addressed, or a specific parenting goal (Kuczynski & De Mol, 2015).

In sum, we examined a longitudinal path from early ecological adversity, a distal risk factor, to later child SR. We conceptualized parental power assertion as a mediating mechanism or a proximal risk factor, accounting for the negative impact of adversity (Nolen-Hoeksema & Watkins, 2011). We further conceptualized parental negative IWMs of the child as moderators accounting for multifinality of trajectories unfolding in the aftermath of adversity. This perspective integrates various developmental contexts, emphasizes continuity and changes across life stages, and highlights the interplay of risk and protective processes, aligning with the bioecological model’s emphasis on dynamics among person, process, context, and time (Bronfenbrenner & Morris, 2007) and with the tenets of developmental psychopathology.

When mapping the developmental path, we assessed ecological adversity impinging on the family when children were infants. Extending previous research, which had commonly assessed parental power assertion and their IWMs only once, we measured both twice – at 16 months and at age 3. Age 16 months is typically considered the time of onset of parental control, but toddler years continue to be replete with conflict, resistance, and common control struggles, making the dynamics between parents and children challenging and complicated. Measuring both IWMs and parenting repeatedly produces a more complete picture of the longitudinal developmental cascades originating in ecological adversity experienced by the family in infancy and it may help pinpoint whether there is a specific window or windows in which IWMs exert their moderating effects. Humphreys and colleagues (2024) explicitly advocated repeated longitudinal assessments of parental IWMs as a goal for the future agenda in research in developmental psychopathology. Investigating repeated measures of mediating and moderating factors can provide a deeper, more developmentally informed understanding of mechanisms and protective factors that enable children from at-risk contexts to develop more adaptive SR skills, and of multifinality in the developmental pathways that can be triggered by early adversity.

First, we examined IWM at 16 months as influencing the path from ecological adversity to parental power assertion at 16 months. Based on past research, we expected that parents facing higher ecological adversity would rely on more power-assertive control, but only if they also had more negative IWMs of the child. Second, we examined negative IWMs at age 3 as potentially influencing the autoregressive path from power assertion at 16 months to power assertion at age 3. Due to the scarcity of research, the examination of the latter effect was exploratory. For example, we considered it possible that for parents with high negative IWMs at 16 months, their elevated power-assertive control would further increase at age 3 if their negative IWMs are also high at age 3. However, we also considered another potential scenario – that once elevated at 16 months, high power-assertive control may remain high regardless of parental negative IWMs at age 3. The latter scenario would imply a particularly lasting significance of the early emerging parental IWMs.

We relied on empirically and theoretically proven measures. We measured ecological adversity using an approach proposed by Kochanska et al., (2007, 2012, 2013), informed by research on established ecological factors that challenge early parenting. Those often include six factors: parental young age (Crugnola et al., 2014; Duncan et al., 2018), low education and low income (Cooper & Stewart, 2021; Dickson et al., 2016), decreasing stability of the family structure (married, cohabitating, single, divorced; Bachman et al., 2011; Lee & McLanahan, 2015), more children (Keenan et al., 2007; Trentacosta et al., 2008), and more stressful recent life events (Abidin, 1992; Reiss et al., 2019). Although often researchers studying ecological adversity score each risk as present or absent and sum them, more fine-grained approaches have been advocated in research on cumulative risks (Burchinal et al., 2008; Evans et al., 2013; Kochanska et al., 2007, 2012). To that effect, we graded the severity of each of those six risk factors, incorporating data for each parent, and summed them into an overall score.

We assessed parents’ IWMs using the Five-Minute Speech Sample interview (FMSS, Bullock & Dishion, 2007). FMSS has become one of the most prominent instruments in developmental psychology, with comprehensive reviews supporting its value, especially the dimension of criticism, or the expression of negative relational schemas regarding the child (Narayan et al., 2012, 2015; Rea et al., 2020; Sher-Censor, 2015; Waller et al., 2012; Weston et al., 2017). FMSS is especially well suited for research informed by attachment theory, as its assessment of the parent’s relational schemas captures both conscious or explicit and sub-conscious or implicit elements, consistent with Bowlby’s construct of IWMs (Bowlby, 1969/1982; Thompson et al., 2022).

We observed parental power assertion in lengthy naturalistic parent-child interactions, designed to be “saturated” with control issues typical for the toddler age. That approach has consistently produced valid and rich measures of parenting behaviors (An & Kochanska, 2022a; An et al., 2022; Kochanska et al., 2001; Kuczynski & Kochanska, 1990), and has been often adopted (e.g., Dahl & Chan, 2017). Finally, we measured child SR using both parents’ reports and observed performance in standard tasks to minimize the method bias and increase the ecological validity of the results (Bank et al., 1990).

We collected all measures from both mother-child and father-child dyads to address the stubborn gap in parenting research, as studies on the relations between ecological adversity and parenting or between parenting and SR – and parenting in general – have predominantly involved mothers only. For instance, a meta-analysis on the associations between parenting and SR reported that fewer than 20% of studies included both mothers and fathers (Karreman et al., 2006). Yet, the importance of fathers’ contributions to children’s development, and specifically in the context of resilience under challenging conditions, has been emphasized (Feldman, 2023).

## Method

### Participants

The current study included 200 typically developing 8-month-old infants (96 girls and 104 boys) born in 2017 and 2018 and their biological parents (mothers and fathers), recruited through flyers and advertisements distributed in various community venues in a U.S. Midwestern region and posts on social media targeting parent groups. The familieswere mostly White,but one orboth parents were not White in 20% of the families. The median household income was $85,000 (SD = $44,530, ranging from $4,000 to $320,000). The levels of parents’ education were as follows: 39% of mothers and 32.5% of fathers had a postgraduate degree, 46.5% of mothers and 43.5% of fathers had an associate or bachelor’s degree, and 14.5% of mothers and 24% of fathers had no more than high school education.

All data were collected during 2–3-hour carefully scripted sessions at home (at age 8 months, *N* = 200, 96 girls, 104 boys) and in naturalistic laboratory settings including a Living Room and a Play Room (at 16 months, *N* = 194, 93 girls, 101 boys, at age 3, *N* = 175, 86 girls, 89 boys, and at age 4.5, *N* = 177, 86 girls, 91 boys), conducted by female experimenters (Es).

Data were coded from recorded videos by multiple teams. Coders used 15% – 20% of cases for reliability, followed by regular realignments. Attrition at 3 and 4.5 years was mostly due to the COVID-19 pandemic, although some families provided online data. No differences were found in most of the measures between families that did and did not return at 4.5 years except for two measures: Families that did not return had higher levels of ecological adversity at 8 months, ΔM = .91, ΔSE = .34, t(27.56) = 2.69, p < .05, and maternal power assertion scores at 16 months, ΔM = .60, ΔSE = .20, t(191) = 3.05, p < .01, compared to those that returned. Parents completed informed consent, and the Institutional Review Board at the University of Iowa approved the study (Children and Parents Study, CAPS, 201701705).

### Measures

#### Ecological adversity, 8 months

To create the index of ecological adversity, we assigned graded risk points for demographic characteristics that have been broadly associated with adversity and developmental disadvantages (0–3, except for relationship status, 0–1; Kochanska et al., 2007, 2012, 2013). The scores were as follows (higher scores indicate higher risk).

- Parental age (for each parent): 20 and younger = 3, ages 21 – 22 = 2, ages 23 – 24 = 1, older than 24 = 0
- Parental education (for each parent): did not complete high school = 3, completed high school = 2, associate’s degree = 1, completed college or beyond = 0
- Family annual income: less than $40,000 = 3, $40,000 – $60,000 = 2, $60,000 – $80,000 = 1, greater than $80,000 = 0
- Number of children: four and more = 3, three = 2, two = 1, one = 0
- Relationship status: not married but living together, or other arrangements = 1, married = 0
- Life stress (from the Parenting Stress Index, PSI, Abidin, 2012, encompassing weighted scores of up to 19 stressful life events during the last year for each parent): 15 and greater = 3, 9 – 14 = 2, 4 – 8 = 1, 3 and lower = 0

The scores were transformed using the proportion of maximum scaling method (Little, 2013) to have a consistent scale from 0 to 1 and summed into a composite score of ecological adversity. The possible range was from 0 to 9. There was no difference between families with girls and boys.

#### Parental power assertion, 16 months and 3 years

Parental power assertion was coded in naturalistic interaction and toy cleanup contexts. At 16 months, the naturalistic interaction contexts (total 15 min) included introduction to the laboratory (when E asked the parent to prohibit the child from touching extremely attractive toys and objects on a low shelf, 5 min), free time (5 min), snack prohibition (when the child, already quite hungry, was asked to wait for a snack, 5 min). The toy cleanup lasted 10 min. At age 3, the naturalistic interaction contexts (total 20 min) included introduction to the laboratory (5 min), snack prohibition (5 min), snack (5 min), and play (5 min). The toy cleanup again lasted 10 min. All contexts were carefully scripted to elicit parents’ typical control behaviors toward the child. We coded parental power assertion for each of the 20s segments (for naturalistic interactions) or 30s segments (for toy cleanups) using the following codes: No control (no interaction, purely social exchange, play), Gentle Guidance (gentle, subtle, playful suggestion or direction), Control (non-forceful, matter-of-fact, relatively assertive control), and Forceful or Harsh Control (negative, power-assertive control often accompanied with hostility, frustration, or threats). The verbal, affective, and physical markers of each code were specified on coding conventions based on extensive past research. Reliability, weighted kappas, ranged from .65 – .86 at 16 months and .61 – .92 at 3 years.

We tallied the instances of each code, created relative scores by dividing each tally by the number of coded segments (for naturalistic interaction only), weighted each score by multiplying No Control by 1, Gentle Guidance by 2, Control by 3, and Forceful or Harsh Control by 4, and added and standardized them. We then created a single power assertion score for each parent by averaging across all the naturalistic contexts and the toy cleanup, with a higher score indicative of higher power assertion. At both time points and for both mother-child and father-child dyads, parents directed more power assertion toward boys than girls, *Ms* = −.25 – −.15, *SDs* = .71 – .82 for girls, *Ms* = .14 – .23, *SDs* = .73 – .87 for boys, *t*(147–188.42) = −4.15 – −2.52, *ps* < .05, .01, or .001. Comparisons of mothers’ and fathers’ scores (prior to standardization) revealed that fathers were more power assertive than mothers when children were 16 months, *M* = 22.89, *SD* = 2.71 for mothers, *M* = 24.42, *SD* = 3.52 for fathers, *t*(185) = −5.24, *p* < .001, but not at 3 years.

#### Parental negative internal working model of the child, 16 months and 3 years

At the end of the session, while the child was in a separate room, E conducted the FMSS interview with the parent, asking them to talk about the child and their relationship with the child for 5 minutes (Bullock & Dishion, 2007). E did not provide any further prompts and engaged in paperwork during the entire interview, consistent with FMSS instructions. The audio-recorded speech was coded by a professional coding team at another university.

We focused on the Criticism scale as reflecting the parent’s negative IWM of the child. It consists of 6 9-point items tapping negative evaluations or messages about the child, such as being critical regarding behavior, traits, or personality of the child, expressing negative humor or sarcasm regarding the child (1 = no evidence, 9 = clear, multiple examples). The average percent agreement among coders was 95.6% at 16 months and 93.6% at 3 years (with 80% being the required standard, Smith et al., 2013; Waller et al., 2012). Additionally, intra-class correlations (ICCs) for the Criticism scale were .75 at 16 months and .80 at 3 years. At 16 months, one of the items was dropped because it had high kurtosis and skewness and lowered internal consistency. We standardized and averaged the remaining 5 items to create a final IWM score (Cronbach’s alphas = .59 for mothers, .52 for fathers). At 3 years, a total of 6 items were standardized and averaged (Cronbach’s alphas = .69 for mothers, .69 for fathers). There were no gender differences. When comparing unstandardized scores between mothers and fathers, mothers had more negative IWM of the child at 16 months, mothers, *M* = 2.19, *SD* = 0.94; fathers, *M* = 1.93, *SD* = 0.79, *t*(185) = 3.22, *p* < .01, and at 3 years, mothers, *M* = 2.12, *SD* = 1.03; fathers, *M* = 1.80, *SD* = 0.84, *t*(149) = 3.31, *p* < .01.

#### Child self-regulation (SR), 4.5 years

We used two measures of child SR: parent-reported SR difficulties and observed SR.

##### Parent-Reported SR Difficulties.

We used mothers’ and fathers’ scores from Children’s Behavior Questionnaire (CBQ; Rothbart et al., 2001). We selected Frustration (6 items, e.g., Gets angry when told s/he has to go to bed) and (reversed) Inhibitory Control (6 items, e.g., Can easily stop an activity when s/he is told “no”), all rated on a 7-point Likert scale (1 = extremely untrue, 7 = extremely true). Cronbach’s alphas of each measure were acceptable: Frustration .75 for mothers, .78 for fathers; Inhibitory Control .62 for mothers, .65 for fathers. The two scales cohered (*r* = .33, *p* < .001), and were standardized and averaged into the final mother- and father-reported SR difficulty scores. Higher scores denote more difficulties in SR. Boys were rated as having more SR difficulties than girls by mothers, *M* = −.24, *SD* = .76 for girls, *M* = .23, *SD* = .80 for boys, *t*(169) = −3.88, *p* < .001, and by fathers, *M* = −.22, *SD* = .85 for girls, *M* = .21, *SD* = .74 for boys, *t*(157) = −3.40, *p* < .001. There were no differences between mothers’ and fathers’ scores.

##### Observed SR.

The tasks to measure active SR performance included Day/Night and Snow/Grass (Carlson & Moses, 2001), Gift Delay – Wrap and Bow, and Gift Delay – Bag (Kochanska et al., 2000). In the Day/Night task, E showed the child two cards, one depicting a nighttime sky and the other depicting a daytime sky, and asked them to point to the nighttime sky when E says “Day” and the daytime sky when E says “Night” for 10 trials (5 trials each for Day and Night). The same procedure was applied to Snow/ Grass. Child answers were coded as 0 = fails to point, 1 = incorrect and never self-corrects, 2 = self-corrects, or 3 = correct on first attempt and doesn’t change mind. We standardized the summed score of Day/Night and Snow/Grass. ICCs ranged from .99 to 1.00.

Gift Delay – Wrap and Bow was performed in mother-child sessions. E brought a loosely wrapped gift and asked the child not to look at the gift while she was wrapping it up. E wrapped the gift noisily for 60 s with her back to the child (Wrap segment). Child behavior was coded as 0 = turns around, doesn’t return fully forward, 1 = turns around but turns back forward, 2 = peeks over shoulder far enough to see wrapping, 3 = turns head to side but less than 90 degrees, or 4 = does not try to peek. Latency to worst transgression was also coded in seconds (60 if never). After 60 s, E said she had forgotten a ribbon for a bow, asked the child to stay in the chair and not to touch or peek inside until she returned, and left the room for 3 min (Bow segment). Child behavior was coded as 0 = opens gift, 1 = lifts up gift, 2 = touches gift but does not lift, or 3 = does not touch gift. Child seat score was also coded as 0 = in seat for less than 30 s, 1 = in seat for morethan30 s butless than 1 min, 2 = in seatformorethan1 minbut less than 2 min, or 3 = in seat for more than 2 min. Latencies to the “worst” violationand to leavingthe seatwere coded inseconds (180sif never). We aggregated the behavior scores, seat scores, and latencies from the two segments into a single score by standardizing and averaging them (Cronbach’s alpha = .74). Reliability, weighted kappa, for the behavior score was .96, and ICC for the latency score was 1.00 for the Wrap segment. Kappas, for behavior and seat scores ranged from .86 to 1.00, and ICC for latencies ranged from .95 to .99 for the Bow segment.

Gift Delay – Bag was an analogous task, administered in father-child sessions. E brought a gift in a bag, said she had forgotten a bow, asked the child to stay in the chair and not to touch or peek in the bag until she returned, and left for 3 min. Child behavior was coded as 0 = removes gift from bag, 1 = puts hand into bag but does not remove gift, 2 = peeks in, with or without touching, 3 = touches bag without peeking, or 4 = does not touch bag or peek. Child seat score and latencies to worst transgression and leaving the seat were coded analogously to Gift Delay – Wrap and Bow. We created a single score by standardizing and averaging the behavior score, seat score, and latencies (Cronbach’s alpha = .66). Kappas for behavior and seat scores were .89 – 1.00, and ICCs for latencies were .89 – 1.00.

We created the observed SR score by aggregating scores across tasks (*rs* = .18 – .53, *ps* < .05 or .001). Higher scores indicate better observed SR. Girls outperformed boys, *M* = .12, *SD* = .75 for girls, *M* = −.16, *SD* = .78 for boys, *t*(155) = 2.24, *p* < .05.

##### Data Aggregation.

Because the two measures, parent-reported SR difficulties and observed SR, were significantly correlated (*r* = −.30 for mothers, *r* = −.28 for fathers, *ps* < .001) and had similar patterns of relations with the predictors (described in the sensitivity analyses below), we averaged them within the mother-child and father-child dyads to simplify the model (having reversed observed SR to denote SR difficulties).

#### Covariates

We covaried children’s gender, their scores on Orienting/ Regulatory Capacity (ORC), assessed at 8 months, and the other parent’s power assertion at age 16 months. ORC, defined by Duration of Orienting, Low Intensity Pleasure, Cuddliness, and Soothability, is often considered a fledgling early form, or antecedent of future SR, which is not typically assessed until after age 18 months. We averaged and standardized the mean of 12 ORC items reported by parents in the Infant Behavior Questionnaire – Very Short Form (Putnam et al., 2014), answered on a 7-point Likert scale. Cronbach’s alphas were .66 for mother-child dyads and .77 for father-child dyads.

## Results

### Descriptive statistics and correlations

Descriptive statistics and the correlations among variables computed in SPSS 29 (IBM Corp, 2023) are presented in Table 1. Higher ecological adversity at 8 months was related to mothers’ higher power assertion and their more negative IWMs at 16 months but not at 3 years. However, no significant correlations were found between ecological adversity and either power assertion or negative IWMs in father-child dyads.

Higher ecological adversity was related to children’s more SR difficulties at 4.5 years in mother-child but not in father-child dyads. Maternal higher power assertion at 16 months and 3 years both correlated with more SR difficulties at 4.5 years. Paternal higher power assertion at 3 years correlated with more SR difficulties at 4.5 years. Maternal and paternal power assertion were moderately stable from 16 months to 3 years.

### Main analysis: Parental negative internal working model of the child as a moderator of the path from ecological adversity to child self-regulation, mediated by parental power assertion

To test the proposed moderated mediation, ecological adversity was modeled as the predictor of child SR difficulties (i.e., combined scores of parent-reported SR difficulties and reversed observed SR) separately in mother-child and father-child dyads. Parental power assertion scores at 16 months and 3 years were modeled as consecutive mediators (an autoregressive path). Parental negative IWM of the child at 16 months was modeled as a moderator of the path from ecological adversity at 8 months to power assertion at 16 months, and negative IWM at 3 years as a moderator of the path from power assertion at 16 months to power assertion at 3 years. Child gender, ORC, and the other parent’s power assertion at 16 months were covaried.

The path models were estimated in Mplus 7 (Muthén & Muthén, 1998–2012) with 5,000-sample bootstrapping, using codes adapted from Stride et al. (2015). Based on Little’s (1988) missing completely at random (MCAR) test, data were considered MCAR in both mother-child, χ^2^(60) = 61.24, *p* = .43, and father-child models, χ^2^(68) = 69.54, *p* = .43. Thus, we used full information maximum likelihood estimator to handle missing data. The significance of mediation was tested using a biascorrected 95% confidence interval (CI). For significant moderation, simple slopes were probed and plotted at the 16^th^ and 84^th^ percentile (Hayes, 2022). Model fit was evaluated using the chisquare, the comparative fit index (CFI), and the root mean square error of approximation (RMSEA) and its 90% CI. Model fit is considered good when the chi-square is not significant, CFI is larger than or equal to .95, and RMSEA is less than or equal to .05 and acceptable when CFI is larger than or equal to .90 and RMSEA is less than or equal to .08 (Hu & Bentler, 1999; Little, 2013).

### Mother-child dyads

**Figure 1.**
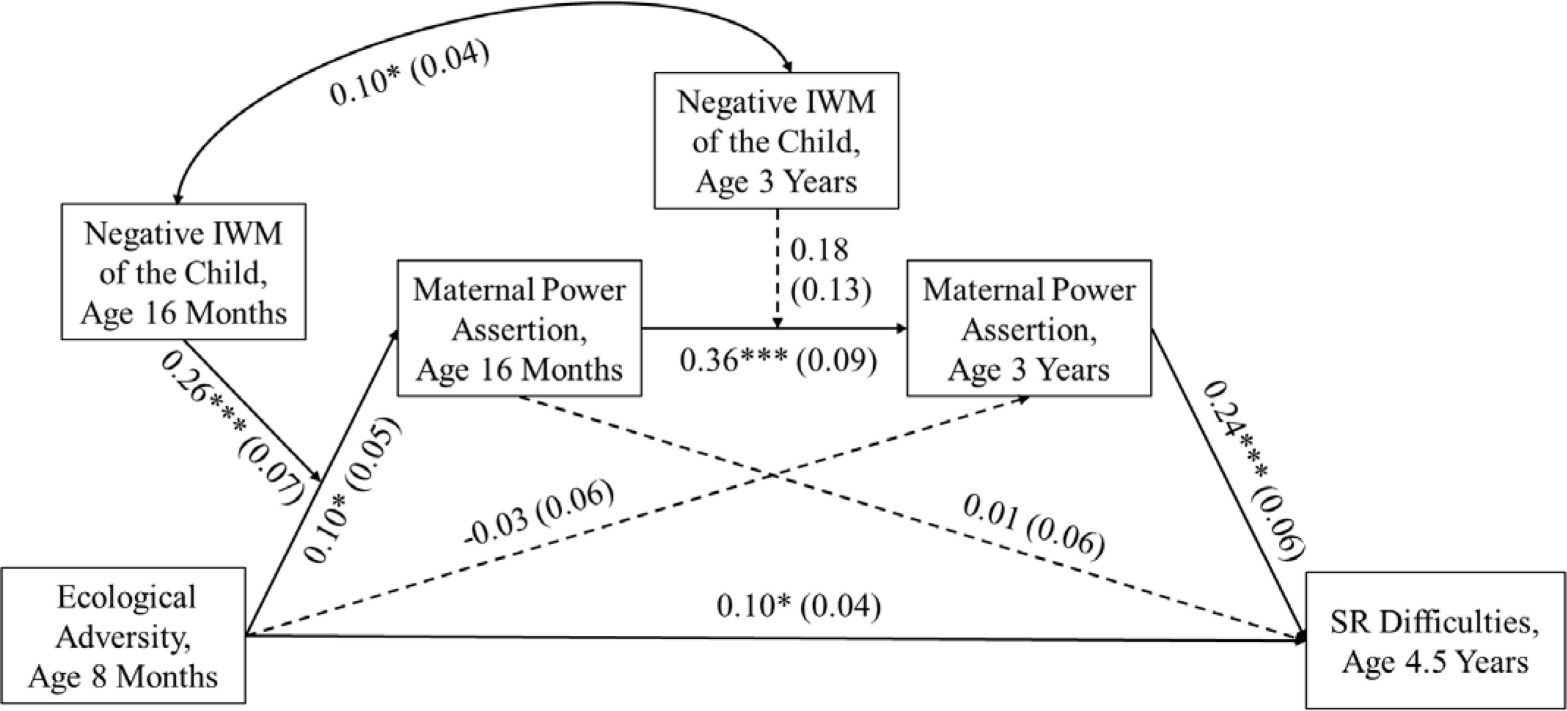
Longitudinal relations from ecological adversity at age 8 months to maternal power assertion at age 16 months and age 3 years to children’s self-regulation at age 4.5 years in mother-child dyads. *Note*. IWM = internal working model, SR = self-regulation. Solid lines represent significant paths and dashed lines represent non-significant paths. Child gender, SR antecedent at age 8 months (Orienting/Regulatory capacity), and paternal power assertion at age 16 months were covaried but not depicted for clarity. Unstandardized coefficients and standard errors (in parentheses) are presented. * *p* < .05. *** *p* < .001. Significant moderated mediation from ecological adversity at age 8 months to SR difficulties at age 4.5 years through maternal power assertion at age 16 months and age 3 years, with mothers’ negative IWM of the child at age 16 months serving as the significant moderator (see Table 2).

The results are presented in Figure 1. First, the direct paths from early ecological adversity to later child SR were significant, such that higher ecological adversity at 8 months was related to more SR difficulties at 4.5 years.

**Figure 2.**
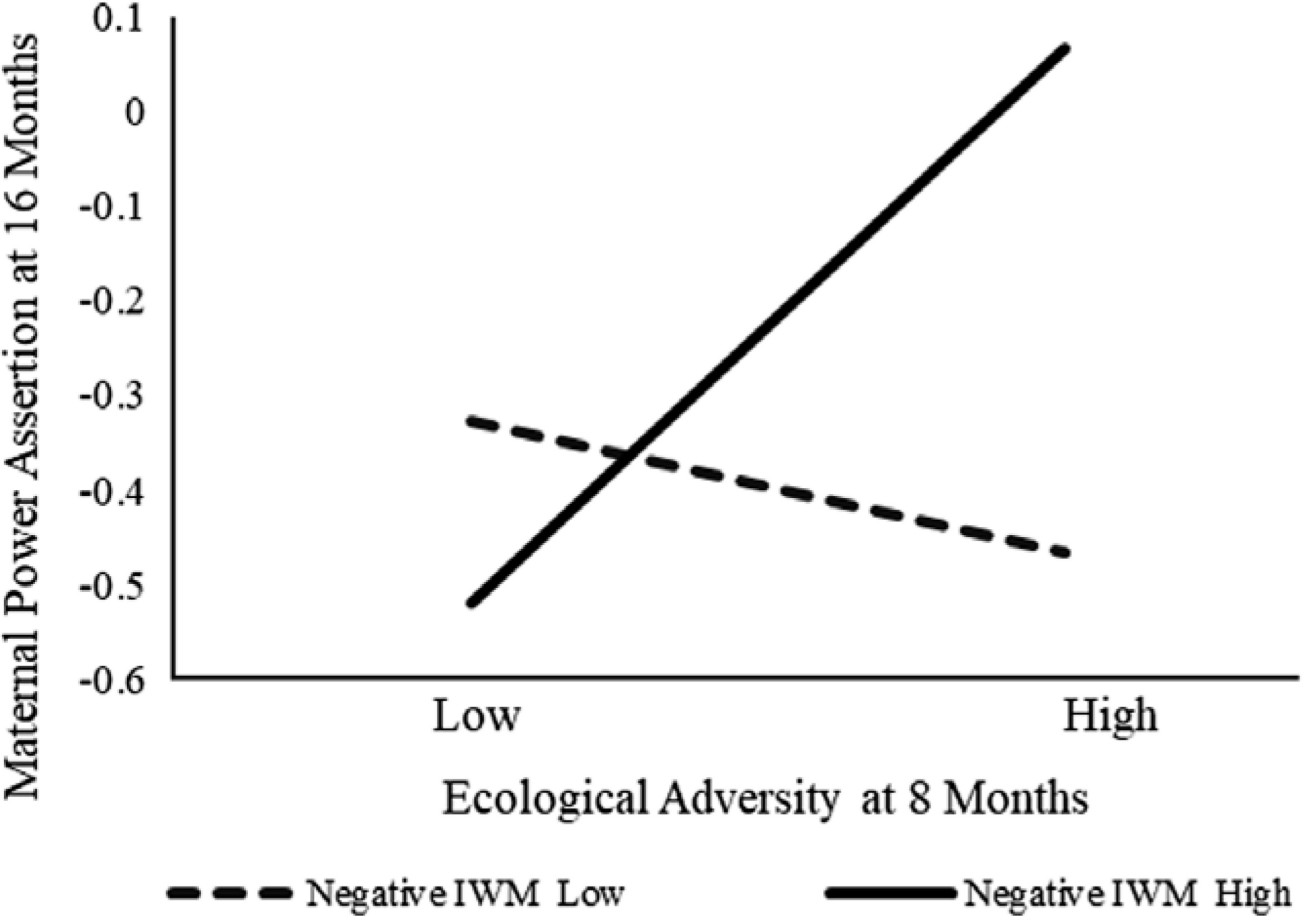
Maternal negative internal working model of the child at age 16 months as a moderator of the relation between ecological adversity at age 8 months and maternal power assertion at age 16 months. *Note*. IWM = internal working model. A simple slope of maternal negative IWM at the high level (84^th^ percentile) was 0.23, *SE* = 0.06, 95% confidence interval [0.11, 0.35], *p* < .001.

Second, we examined the paths from the predictor to the mediators as well as the moderated effects of maternal negative IWMs. Ecological adversity at 8 months was significantly related to maternal power assertion at 16 months but not to maternal power assertion at 3 years. Additionally, as expected, the former path was moderated by maternal negative IWMs of the child at 16 months. Mothers who experienced higher ecologicaladversity when their child was an infant relied on more power assertion at 16 months, but only if they also had highly negative IWMs of the child, *B* = 0.23, *SE* = 0.06, 95% CI [0.11, 0.35], *p* < .001. Simple slopes are depicted in Figure 2.

Higher maternal power assertion at 16 months was related to higher power assertion at 3 years, but this path was not moderated by maternal negative IWM of the child at 3 years. In other words, the stability of maternal power assertion from 16 months to 3 years was not contingent on the mothers’ negative IWMs of the child.

Third, we examined the paths from the mediators to the outcomes. Higher maternal power assertion at 3 years was related to children’s more SR difficulties at 4.5 years. However, there were no significant paths from maternal power assertion at 16 months to child SR difficulties.

Finally, fourth, we tested the significance of moderated mediation for the entire cascade from early ecological adversity to SR at preschool age: We examined if the paths from ecological adversity in infancy to child SR through maternal power assertion at 16 months and at 3 years were significantly different for mothers differing in their negative IWMs of the child. Maternal negative IWMs of the child at 16 months moderated the mediated path from early ecological adversity to mother-reported SR difficulties through maternal power assertion at 16 months and then 3 years. When maternal negative IWM of the child was high at 16 months, higher ecological adversity in infancy initiated a cascade to first, more power assertion at 16 months, and next, to more power assertion at age 3, and ultimately, to more mother-reported SR, regardless of the level of maternal negative IWM of the child at 3 years.

We include Table 2 to illustrate the presence or absence of moderated mediation for the entire path from ecological adversity at 8 months to SR at 4.5 years for mothers with high (16^th^ percentile) or low (84^th^ percentile) negative IWMs of the child at both times when IWMs were assessed. Although recall that only IWMs at 16 months exerted statistically significant moderating impacts and unique moderation by maternal negative IWM at 3 years was not significant, the examination of effect sizes for the moderated mediation is informative. The moderating effect seemed bolstered when maternal negative IWMs of the child at both 16 months and at 3 years were high, although the difference in effects narrowly missed statistical significance, ΔB = 0.013, SE = 0.012, 95% CI [−0.002, 0.049].

In sum, higher ecological adversity parents experienced when their child was an infant might be detrimental for later parenting and subsequent child SR, especially when mothers had an early formed, highly negative IWM of the child. However, the negative effects could be buffered when mothers had less negative IWM of the child.

#### Father-child dyads.

**Figure 3.**
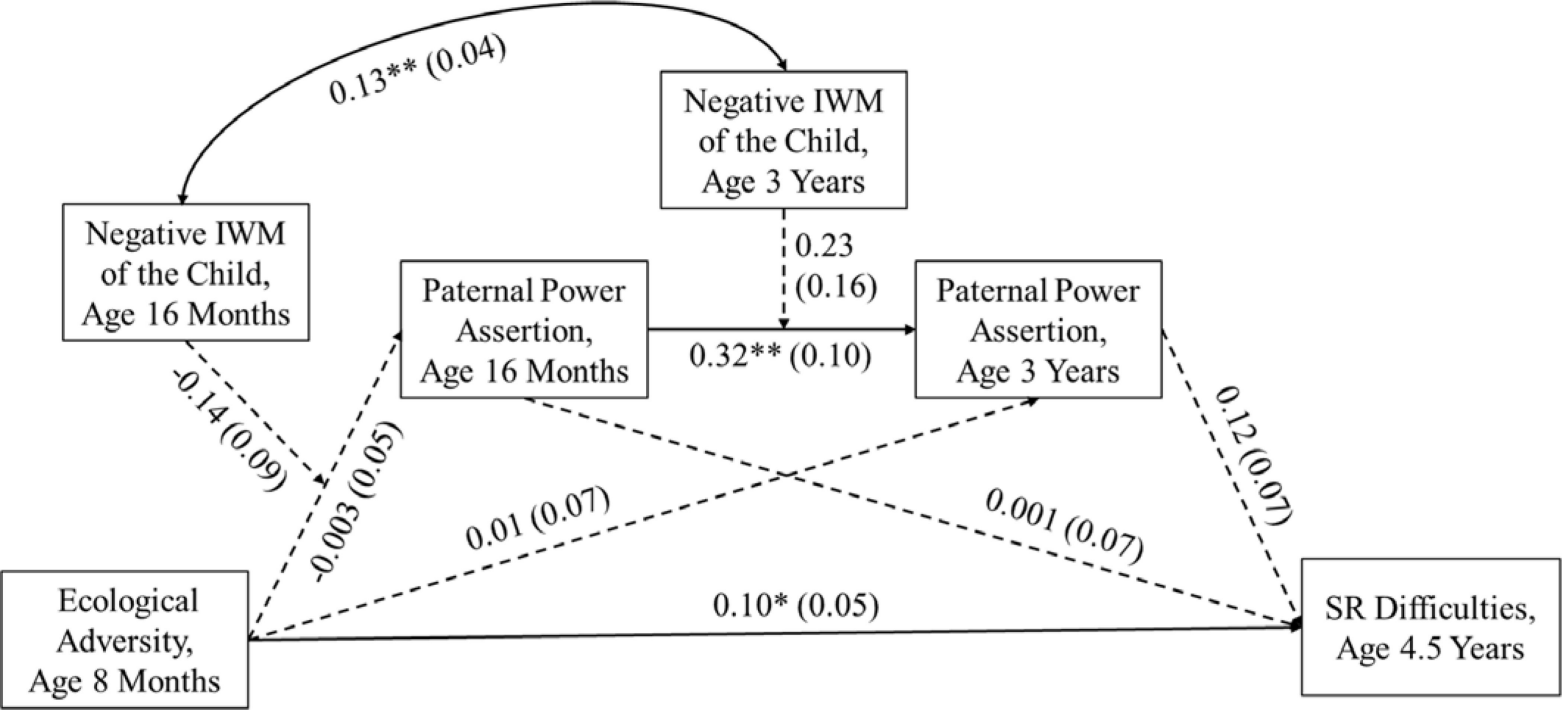
Longitudinal relations from ecological adversity at age 8 months to paternal power assertion at age 16 months and age 3 years to children’s self-regulation at age 4.5 years in father-child dyads. *Note*. IWM = internal working model, SR = self-regulation. Solid lines represent significant paths and dashed lines represent non-significant paths. Child gender, SR antecedent at 8months(Orienting/Regulatory capacity), and maternal power assertion at age 16 months were covaried but not depicted for clarity. Unstandardized coefficients and standard errors (in parentheses) are presented. * *p* < .05. ** *p* < .01. No significant moderated mediation was found.

Relatively few significant paths were found in father-child dyads. The results are in Figure 3. As for mothers, higher ecological adversity at 8 months was directly related to more SR difficulties at 4.5 years and paternal power assertion was stable from 16 months to 3 years. Paternal power assertion at 16 months and 3 years did not predict children’s SR difficulties. In contrast to mother-child dyads, we found no evidence of significant mediation through power assertion or moderation by negative IWM of the child.

#### Sensitivity analyses.

We conducted additional analyses with separate SR variables – parent-reported SR difficulties and observed SR – modeled as child outcomes. The results of the original models were essentially replicated in both mother-child and father-child dyads. We found significant moderated mediation in mother-child dyads, from higher early ecological adversity to poorer observed SR through higher maternal power assertion for mothers with more negative IWM of the child at 16 months. The moderated mediation on mother-reported SR difficulties yielded similar patterns. The results are in Supplement Table S1. We found no significant moderated mediation in father-child dyads.

Additionally, we estimated alternative models that included parental power assertion and negative IWM of the child at 16 months only (without the respective measures at 3 years). The findings are in Supplement Table S2, Figure S1. In both dyads, our key hypothesized model (one including power assertion and IWM at both 16 months and age 3) was supported, given that the chi-square difference was not significant between the two models and other fit indices were similar. Consequently, our conclusions did not change.

## Discussion

Early exposure to ecological adversity affecting families is universally seen as potentially transdiagnostic risk factor, detrimental to children’s future outcomes. However, understanding specific mechanism or mechanisms mediating such effects along the developmental trajectory is incomplete. Understanding moderating factors that explain multifinality in those trajectories or identifying parents who are and who are not negatively affected is likewise incomplete. Additionally, very little is known about such moderated mediation processes in father-child relationships.

We traced the longitudinal paths from ecological adversity in infancy to one important developmental outcome – self-regulatory difficulties – at preschool age in both mother-child and father-child dyads. We proposed parental power assertion and negative IWMs of the child in toddlerhood as mediators and moderators of those paths, respectively. Given that toddlerhood is a period during which parents and children often navigate control challenges, we assessed parental power assertion and negative IWMs at the approximate times of onset and offset of the toddler age (at 16 months and at 3 years). We included those two developmental windows to better understand the timing when moderating factors may exert their impacts.

In mother-child dyads, we supported our conceptual model. Power-assertive parenting mediated the path from early ecological adversity to later child SR, and mothers’ negative IWMs of the child moderated the mediated path. For mothers who had highly negative IWMs of the child, higher ecological adversity when children were infants predicted more power-assertive control in toddlerhood, which in turn predicted poorer SR at preschool age. This mediated relation, however, was absent for mothers whose IWMs of the child were less negative. These results are consistent with previous findings regarding significant indirect relations from multiple risk factors to behavioral outcomes and executive function through parenting (FLP Key Investigators, 2013; Rhoades et al., 2011); however, we add to that previous research. Due to the inclusion of parental IWMs of the child, our findings are more nuanced, in that they specify for whom the relations are present, consistent with recent research that considered a critical moderating role of parental representations (An & Kochanska, 2022b, Kochanska & An, 2024a, 2024b; Kochanska et al., 2019). The findings highlight the complex interplay between individuals and contexts, consistent with bioecological models.

The timing of developmental events and potential sensitive periods have always been core and compelling topics in developmental psychopathology. The findings provide initial insights into specific developmental windows critical for the effects due to maternal negative IWMs. Maternal negative IWMs moderated the path from ecological adversity to maternal power assertion at 16 months, but not the autoregressive path for power assertion across the toddler age (16 months to age 3). These findings are new and important, as they illustrate how maternal early implicit negative schemas regarding the child, formed at the end of the first year, can impact harsh parenting for mothers coping with adverse circumstances. Mothers with higher negative IWMs of the child, when faced with stressful circumstances, may be more vulnerable to environmental risks and get easily distressed. In turn, higher stress can increase the likelihood of harsh and coercive parenting. Importantly, once harsh parenting is elevated, it remains high through the toddler age regardless of maternal future perceptions of the child.

Consistent with the tenets of developmental psychopathology, these findings have implications for both basic and translational research. They support the attachment theory’s premise that early experiences can be “privileged” in development (Sroufe, 2009) and that they play an especially important role in developmental trajectories triggered by adversity (Gee & Cohodes, 2023). Maternal negative IWMs of their children, formed throughout the first year, prior to the onset of control, have significant consequences for mothers’ discipline style once control issues become salient, and their impactful detrimental legacy continues through preschool age, affecting children’s SR skills as they face the important transition to kindergarten.

The findings also have implications for the question of which risk factors are modifiable. Although maternal negative schemas at age 3 per se no longer impacted parenting, the entire detrimental trajectory from ecological adversity to harsh parenting to children’s poor SR appeared most pronounced for mothers whose negative IWMs were high at both assessments (note that this inference should be treated with caution and as a preliminary but promising lead, see Table 2). In other words, our key findings indicate that maternal power assertion, elevated at 16 months for mothers with highly negative IWMs, remained stable; however, it is possible that negative IWMs may continue to matter in the associations among adversity, parenting, and child SR throughout the early years. This pattern of findings highlights the need for intervention for parents coping with difficult circumstances as early as infancy and suggests that such early interventions may be most beneficial. Several interventions addressing maternal IWMs have proved successful (Borelli et al., 2023; Camoirano, 2017; Suchman et al., 2017). Important from a prevention point of view, lower levels of negative IWMs can buffer the negative effects of early ecological adversity to later SR by weakening or severing the link between adversity and harsh parenting at the beginning of the second year. Interventions later in development, however, past age 3, may miss the important window and be less effective.

In the father-child model, the individual paths showed similar patterns as in the mother-child model. However, we did not replicate any significant moderation or moderated mediation found in mother-child dyads. It is inconsistent with the previous results that showed a significant moderating effect of paternal mentalization on the relations between family income and autonomy support (Buttitta et al., 2019). Whereas the latter study relied on a single source of adversity (i.e., low income), we combined several sources of demographic and environmental risks, which might render the interaction between ecological adversity and paternal mentalization more complicated.

The discrepant findings between mother-child and father-child relationships suggest potential differences in the associations among ecological adversity, parental negative IWMs, and power assertion. Mothers’ greater investment in child care can explain, in part, different findings for the two parent-child relationships. In our sample, at the entry to the study, mothers spent approximately 59 hours per week on average with the child, while fathers spent 35 hours. Thus, mothers’ parenting may have been more vulnerable to environmental risks compared to fathers.

In both mother-child and father-child relationships, the direct effects of early ecological adversity on child future SR remained significant even after accounting for the mediation effects of parenting and moderating effects of the IWMs. There are several possible interpretations of these findings, involving constructs we did not measure. SR does have a genetic and neural basis (Blair, 2010; Bridgett et al., 2015; Conradt et al., 2015; Perone et al., 2018), shared between the parent and the child. Parents whose own self-regulatory capacities are poor are likely to be over-represented in high-risk ecologies, as SR robustly predicts multiple dimensions that are markers of such ecologies (low education, young age at childbirth, unstable family structures, etc., Moffitt et al., 2011, 2013). Thus, ecological, genetic, socialization, and experiential factors all work in concert, accounting for developmental trajectories of children’s SR (Deater-Deckard, 2014).

This work has several strengths. We studied both mother- and father-child dyads, and employed well-established measures, such as a fine-grained index to assess cumulative ecological adversity, observed behavioral data for parental power assertion, and parent-reported and observed measures for child SR. The FMSS interviews to capture parental implicit negative relational schemas or IWMs of the child were especially valuable, as many parents may feel reluctant to share – or may have difficulty accessing cognitively – their negative emotions and views explicitly. Yet, such implicit relational schema exerts a significant impact on their parenting. We further believe that our investigation of developmental timing of effects of parental IWMs was an innovative aspect of this work and, although preliminary, provided leads for future research on this question.

The current study had limitations. Ethnic diversity was limited (although recall that 20% of families were not “White alone”). Parents and children were mostly from low-risk families. Although ecological adversity data were normally distributed, its levels were low – likely lower than adversity in the overall population. Parentchild relationships were generally adaptive and harmonious, with power assertion used relatively rarely. Parents’ Criticism scores in FMSS were also low.

In addition, it can be argued that our ecological adversity construct was in part arbitrary. We focused on ecological adversity in terms of sociodemographic risks, but other dimensions of adversity could certainly be considered. One example is intergenerational trauma, including the parent’s experience of maltreatment, which can uniquely impact parenting (Assink et al., 2018). As well, underrepresented racial or ethnic groups are likely to experience historical trauma and have higher rates of related symptoms such as post-traumatic stress disorder (O’Neill et al., 2016; Pole et al., 2008). Parental mental or physical illness is another possible dimension of early adversity, as are environmental factors such as the quality of neighborhoods (Ursache et al., 2022) and early childhood education (Kangas et al., 2015; Williford et al., 2013).

Multiple future directions of research can extend this work. Aspects of parenting other than power-assertive control can serve as proximal risk factors (e.g., parental unresponsiveness or emotional unavailability, inconsistent discipline, intrusiveness), and they can be potential candidates accounting for the link between ecological adversity and SR. In addition, individual differences in children’s emotion regulation processes can be substantially involved in the pathways from the ecological context to children’s regulatory skills, as proposed in neuroecobiological models. In those models, features of the ecological context are seen as influencing the child’s emotion regulation, which then serves as a mediator linking the context with SR (Liew et al., 2023). Further, children with certain biologically based traits may be more affected than others by ecological adversity and by the quality of parenting they receive, consistent with differential susceptibility models (Belsky & Pluess, 2009). Thus, future studies with multiple mediators or moderators from different systemic levels – from biological markers of both the parent and the child to measures of individuals’ interactions to relationship-based constructs to indices of broader contexts – would provide a more comprehensive understanding regarding a complex, multi-level interplay among person, process, context, and time factors in the development of SR in children in families that experience early adversity (Bronfenbrenner & Morris, 2007; Masten et al., 2023).

Consequently, future research would benefit from including families coping with more extreme adversity, including parental psychopathology, traumatic experiences, chaos, single parenting, poverty, severe deprivation, displacement, and other risks, and engaging in highly dysfunctional parenting, including child maltreatment. More robust indirect effects are expected with more diverse samples including high-risk families, which can emphasize the important role of early adversity more strongly (Taraban & Shaw, 2018). For instance, in a study with Head Start families, harsh discipline mediated the link between socioeconomic adversity and child behavior problems (Baker & Brooks-Gunn, 2020). Thus, if high-risk families are included, non-significant paths in the current study (e.g., the relations between parental power assertion and parent-reported SR difficulties, or relations between adversity and parenting) may likely become significant, making the overall mediation more robust. Of note, in a past study of highly stressed, high-risk, low-income, loweducation, and ethnically diverse mothers and toddlers, our ecological adversity index was indeed robustly associated with observed parenting (Kochanska et al., 2012, 2013). Nevertheless, even in the relatively low-risk, well-functioning families in the current study, we were able to trace a significant negative path from early ecological adversity to later parenting and to child SR, and the detrimental effects of early parental negative mentalization regarding the child, highlighting the critical role of early experiences for future developmental trajectories.

## Supplementary Material

1

## Figures and Tables

**Table 1.** Descriptive statistics of and correlations among study variables

Construct	1	2	3	4	5	6
1. Ecological adversity, age 8 Months	−	.22**	.02	.26***	.01	.16*
2. Parental power assertion, age 16 months	.08	.35***	.38***	.22**	.04	.22**
3. Parental power assertion, age 3 years	.04	.44***	.55***	.11	.16	.35***
4. Parental negative IWM, age 16 months	-.03	.16*	.20*	.13	.25**	.17*
5. Parental negative IWM, age 3 years	.01	.20*	.30***	.34***	.24**	.14
6. Child SR difficulties, age 4.5 years	.10	.14	.25**	.12	.10	.73***
*M*	2.02	0.00 0.00	0.00 0.00	0.00 0.00	0.00 0.00	0.01 0.01
*SD*	1.26	0.83 0.79	0.83 0.86	0.61 0.59	0.63 0.63	0.65 0.69
*N*	200	193 186	157 149	194 186	167 153	174 169

*Note*. IWM = internal working model. SR = self-regulation. Correlations for mother-child dyads are above the diagonal, and correlations for father-child dyads are below the diagonal. Values on the diagonal represent correlations for the variables across mother-child dyads and father-child dyads. For mean, standard deviation, and number of observations, the upper and lower values are from mother-child and father-child dyads, respectively.

* *p* < .05.

** *p* < .01.

*** *p* < .001.

**Table 2.** Moderated mediation from ecological adversity at age 8 months to maternal power assertion at age 16 months and age 3 years to children’s self-regulation at age 4.5 years: Moderation by maternal negative internal working model at age 16 months and at age 3 years

Outcome Variable	Maternal negative internal working model		*B (SE)*	95% Confidence Interval
	At 16 Months	At 3 Years		
Self-regulation difficulties	**High**	**Low**	**0.013 (0.008)**	**0.001, 0.035**
	**High**	**High**	**0.026 (0.012)**	**0.009, 0.058**
	Low	Low	−0.003 (0.005)	−0.016, 0.003
	Low	High	−0.006 (0.008)	−0.025, 0.008

*Note*. The bolded paths depict moderated mediation present for the entire trajectory from ecological adversity to self-regulation. The path was significant for mothers with high negative IMW of the child at 16 months. Note that although maternal negative internal working model (IWM) at 16 months (but not at 3 years) was the significant moderator, the moderated mediation effect appeared bolstered when maternal negative IWM was high at both 16 months and 3 years.
