# Supplementary material for: Multifinality in pathways from early ecological adversity to children’s future self-regulation: Elucidating mechanisms, moderators, and their developmental timing
Source: Dev Psychopathol. Author manuscript; Available in PMC 2025 Sep 21. (PMC12353323; doi:10.1017/S0954579425000148)
Supplement: 1 [file NIHMS2059750-supplement-1.docx]

**Table S1**

*Moderated Mediation from Ecological Adversity at Age 8 Months to Maternal Power Assertion at Age 16 Months and Age 3 Years to Children’s Self-Regulation at Age 4.5 Years: Moderation by Maternal Negative Internal Working Model at Age 16 Months and at Age 3 Years*

| Outcome Variable | Maternal Negative Internal Working Model | | *B* (*SE*) | 95%  Confidence Interval |
| --- | --- | --- | --- | --- |
|  | At 16 Months | At 3 Years |  |  |
| Parent-Reported  Self-Regulation Difficulties | **High** | **Low** | **0.011 (0.008)** | **0.001, 0.034** |
|  | **High** | **High** | **0.020 (0.010)** | **0.005, 0.047** |
|  | Low | Low | -0.003 (0.004) | -0.015, 0.002 |
|  | Low | High | -0.005 (0.006) | -0.021, 0.005 |
| Observed  Self-Regulation | **High** | **Low** | **-0.015 (0.010)** | **-0.040, -0.001** |
|  | **High** | **High** | **-0.027 (0.014)** | **-0.065, -0.009** |
|  | Low | Low | 0.004 (0.005) | -0.003, 0.019 |
|  | Low | High | 0.007 (0.009) | -0.008, 0.028 |

*Note.* The bolded paths depict moderated mediation present for the entire trajectory from ecological adversity to self-regulation.

**Table S2**

*Model Fit Indices of Hypothesized and Alternative Models*

|  | | χ^2^(*df*) | ∆χ^2^(∆*df*) | | CFI | RMSEA [90% CI] |
| --- | --- | --- | --- | --- | --- | --- |
| Mother-Child Dyads | | | | | | |
| **Hypothesized Model** | 19.98(12) | | | - | .98 | .06 [.00, .10] |
| Alternative Model | 8.34(5) | | | 11.64(7) | .99 | .06 [.00, .12] |
| Father-Child Dyads | | | | | | |
| **Hypothesized Model** | | 25.20(12)^*^ | - | | .97 | .07 [.03, .12] |
| Alternative Model | | 15.16(5)^**^ | 10.05(7) | | .97 | .10 [.05, .16] |

Note. Alternative Model = Moderated mediation model with parental power assertion and negative internal working model (IWM) at 16 months only as a mediator and moderator, respectively (parental power assertion and IWM at age 3 excluded). Preferred models are in bold.

^*^ *p* < .05. ^**^ *p* < .01.

**Figure S1**

*Longitudinal Relations from Ecological Adversity at Age 8 Months to Parental Power Assertion at Age 16 Months to Children’s Self-Regulation at Age 4.5 Years*

A


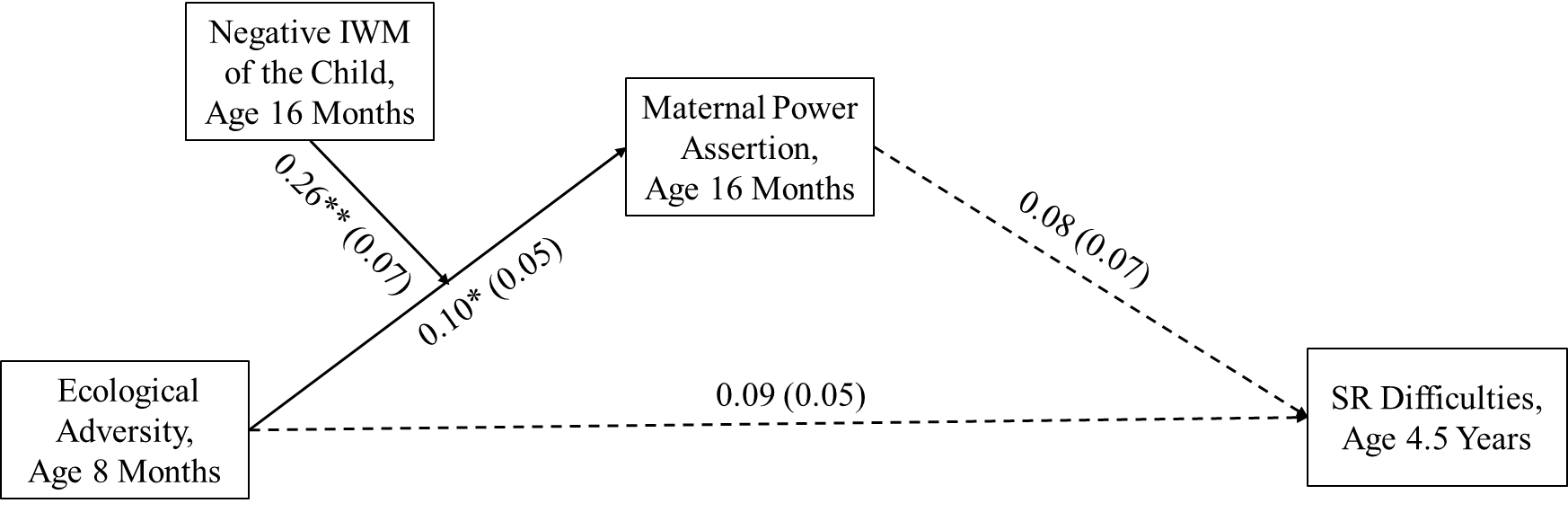


B

*
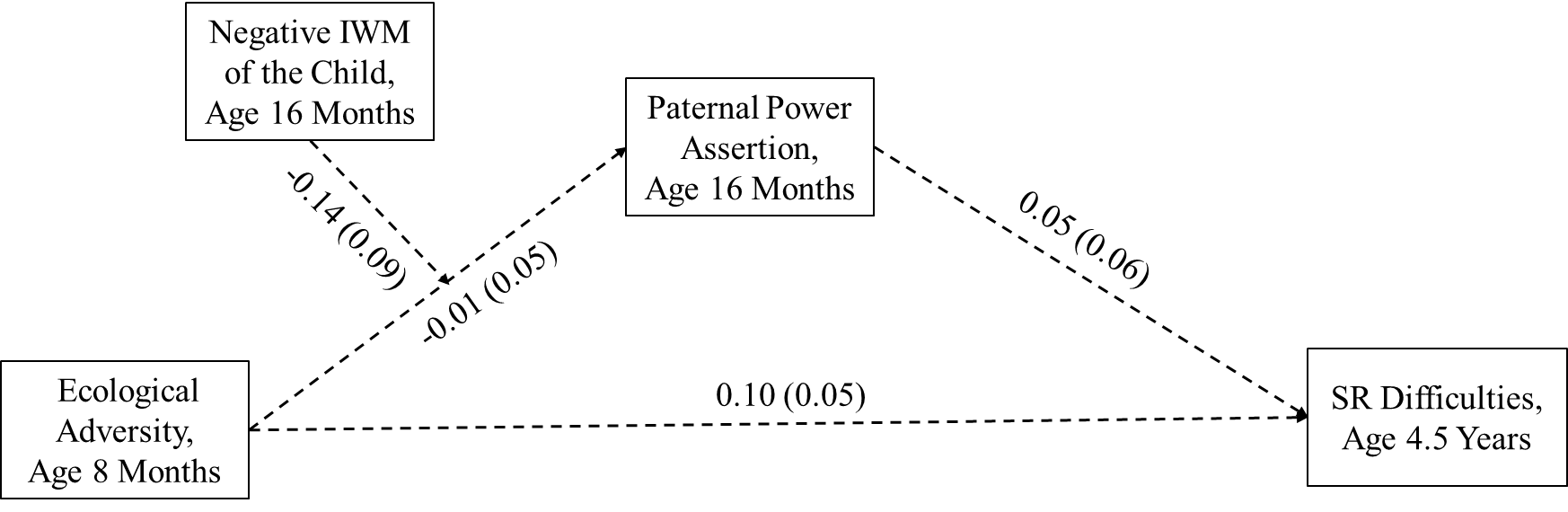
*

*Note.* A = mother-child dyads. B = father-child dyads. IWM = internal working model. SR = self-regulation. Solid lines represent significant paths and dashed lines represent non-significant paths. Child gender, SR antecedent at 8 months (Orienting/Regulatory Capacity), and the other parent’s power assertion at 16 months were covaried but not depicted for clarity. Unstandardized coefficients and standard errors (in parentheses) are presented. ^*^ *p* < .05. ^**^ *p* < .01.
